# Sleep and Performance during a Preseason in Elite Rugby Union Athletes

**DOI:** 10.3390/ijerph18094612

**Published:** 2021-04-27

**Authors:** Angus R. Teece, Christos K. Argus, Nicholas Gill, Martyn Beaven, Ian C. Dunican, Matthew W. Driller

**Affiliations:** 1 Te Huataki Waiora School of Health, University of Waikato, Hamilton 3216, New Zealand; nicholas.gill@nzrugby.co.nz (N.G.); martyn.beaven@waikato.ac.nz (M.B.); 2 Chiefs Rugby Club, Hamilton 3214, New Zealand; christosa@chiefs.co.nz; 3 New Zealand Rugby, Wellington 6011, New Zealand; 4 Centre for Sleep Science, University of Western Australia, Perth 6009, Australia; ian.dunican@meliusconsulting.com.au; 5 Sport and Exercise Science, School of Allied Health, Human Services and Sport, La Trobe University, Melbourne 3086, Australia; M.Driller@latrobe.edu.au

**Keywords:** sleep, athletic performance, rugby union, skinfolds, bronco, preseason

## Abstract

Background: Preseason training optimises adaptations in the physical qualities required in rugby union athletes. Sleep can be compromised during periods of intensified training. Therefore, we investigated the relationship between sleep quantity and changes in physical performance over a preseason phase in professional rugby union athletes. Methods: Twenty-nine professional rugby union athletes (Mean ± SD, age: 23 ± 3 years) had their sleep duration monitored for 3 weeks using wrist actigraphy. Strength and speed were assessed at baseline and at week 3. Aerobic capacity and body composition were assessed at baseline, at week 3 and at week 5. Participants were stratified into 2 groups for analysis: <7 h 30 min sleep per night (LOW, *n* = 15) and >7 h 30 min sleep per night (HIGH, *n* = 14). Results: A significant group x time interaction was determined for aerobic capacity (*p* = 0.02, *d* = 1.25) at week 3 and for skinfolds at week 3 (*p* < 0.01, *d* = 0.58) and at week 5 (*p* = 0.02, *d* = 0.92), in favour of the HIGH sleep group. No differences were evident between groups for strength or speed measures (*p* ≥ 0.05). Conclusion: This study highlights that longer sleep duration during the preseason may assist in enhancing physical qualities including aerobic capacity and body composition in elite rugby union athletes.

## 1. Introduction

Repeated performance is underpinned by an athlete’s ability to recover from multiple physical and psychological stressors [1,2]. Thus, optimal recovery is essential to maximise an elite athlete’s performance [3,4]. Elite athletes utilise numerous recovery strategies [5]; however, sleep is perceived by athletes and researchers to be the most important recovery tool available [2]. Prior research has shown that sleep duration can be compromised during periods of high training demands, such as those experienced during preseason training [6,7,8,9]. Decreased sleep duration can have detrimental effects on athletic performance through impaired cognitive performance, endocrine, mood and metabolic function [10,11]. However, to date, there has been limited research investigating the relationship between sleep and changes in physical performance markers during periods of increased training demands in team sport athletes. 

In rugby union, preseason training aims to enhance physical capacities, including power, speed, strength, aerobic and anaerobic fitness and body composition [12,13,14]. To achieve this, athletes are required to train multiple times a day over consecutive days, which results in a period of intensified training. Research has highlighted that sleep can be negatively affected during intensified training periods such as those seen in the preseason [7,8,9,15]. In addition to this, rugby union athletes have previously been reported as having higher instances of sleep disorders and excessive sleepiness [16]. Therefore, sleep may also be a consideration when factoring in player loads during an intensified training period [17]. While not in rugby union, Killer and colleagues [7] reported a progressive decline in sleep efficiency due to increased wake bouts and movement time during sleep throughout a nine-day intensified training period in highly trained cyclists. Additionally, Thornton and colleagues reported that increases in training volume (e.g., training duration, total distance and high-speed running) could decrease sleep duration and efficiency during periods of intensified training in elite rugby league athletes [9,15]. Increases in training volume have been previously linked to greater muscle soreness [18]. Increases in muscle soreness have been suggested to affect sleep continuity resulting in an increased number of awakenings per night, potentially affecting sleep duration [8].

A reduction in sleep efficiency and duration seen during intensified training periods may be detrimental to maximising physiological adaptation and muscle recovery [19]. Researchers have suggested that reductions in sleep duration may inhibit muscle growth and recovery [19] and lead to a catabolic environment [20,21] with the potential to attenuate muscular adaptation associated with resistance training [10,20,22]. Reductions in sleep duration are also known to lead to an imbalance in hormones (i.e., Leptin and Grehlin) necessary for the control of appetite, satiety, which has been associated with decreases in resting metabolic rate and increased hunger, and caloric consumption and weight gain [19,21,23], which may impact individual athletes depending on the body composition requirements of the sport and or playing position.

In contrast to the reported adverse effects of a reduction in sleep, increasing sleep duration may be beneficial for moderating stress responses and performance during periods of intensified training [24]. Swinbourne and colleagues [24] investigated the effects of three weeks of sleep extension (>8 h) on sleep, stress markers and performance in highly trained male rugby union athletes and observed a mean improvement of 4.3% in reaction time and a reduction of 18.7% in cortisol levels. The findings reported by Swinbourne and colleagues [24] suggest that longer sleep duration during the preseason phase in rugby union may assist in moderating stress responses, potentially supporting optimal adaptive outcomes.

To the best of the authors’ knowledge, there are no studies investigating the relationship between sleep and changes in physical performance markers, including strength, speed, fitness and body composition over an intensified micro-cycle of training (e.g., 3–5 weeks) in elite rugby union athletes. Therefore, the current study aimed to examine the relationship between sleep and physical performance changes throughout a preseason training phase in a group of professional rugby union athletes.

## 2. Materials and Methods

### 2.1. Participants

A total of 29 professional rugby union athletes (mean ± SD; age, 23 ± 3 years; body mass, 104.9 ± 10.7 kg; stature, 187.0 ± 6.9 cm) from a New Zealand Super Rugby team volunteered to participate in the current study. The Super Rugby competition is the premier professional club rugby competition in the southern hemisphere and involves 15 teams from New Zealand, Australia, South Africa, Argentina and Japan. All athletes undertook a 3-week off-season training program prior to the study to ensure athletes arrived at the start of the preseason in appropriate physical condition to meet the subsequent training demands. Written informed consent was obtained from participants, and ethical approval (Ethics number: FEDU066/16) was obtained from the Institutions Research Ethics committee.

### 2.2. Research Design

A pre-post observational study design was utilised for the current investigation. Markers of strength, aerobic fitness, speed and body composition were collected across several sessions throughout the study period. Their relationship to sleep metrics was examined across a five-week training period. Sleep groups were split retrospectively based on average nightly sleep duration. The LOW sleep group displayed an average sleep duration of 6 h 55 min, less than the 7 h, which has been deemed to demonstrate “sleep loss” [25]. Conversely, the HIGH sleep group displayed an average sleep duration of 7 h 49 min, which lies within the recommended sleep duration of 7–9 h in adult populations [26]. The athletes completed a three-week training block during the preseason phase of the competition. Each week within this phase comprised of four training days, which consisted of two speed sessions, four gym-based resistance training sessions, five conditioning sessions and eight rugby specific sessions per week. The 3-week training period was followed by a 2-week maintenance training block, which consisted of three gym-based resistance training sessions and four conditioning sessions per week. All training programs were designed and monitored by the team’s strength and conditioning and coaching staff. Increases in the on-field training load, assessed by GPS, was planned across the three weeks as follows: Week 1—22 km ± 3 km (8 sessions); Week 2—30 km ± 4.5 km and an 8 km hill walk (11 sessions); Week 3—39 km ± 5.8 km (11 sessions).

### 2.3. Procedures

#### 2.3.1. Sleep Monitoring

Quantitative sleep measures were collected throughout the study via the use of wrist actigraphy (ReadiBand^TM^, Fatigue Science, Vancouver, BC, Canada). Athletes were required to wear a wrist actigraph on either wrist [27] throughout the entire 3-week training block and were encouraged to wear the actigraph at all times except during contact training. Athletes were asked to maintain their regular sleep routines throughout the study. For the duration of the study, athletes slept in their residential homes. At the beginning of each day, actigraphy data were wirelessly downloaded to an iPad, and then analysed by online software (16 Hz sampling rate; ReadiBand^TM^, Fatigue Science, Vancouver, BC, Canada). Athletes were blinded to their sleep results throughout the duration of the study. The raw activity data were translated into sleep–wake indices including total sleep time (TST), sleep efficiency (SE%), sleep latency (SL), wake after sleep onset (WASO) and wake episodes (WE). The ReadiBand has been validated against polysomnography (PSG) and has been deemed to be acceptable with an approximately 90% agreement in total sleep duration when compared to PSG [28]. The inter-device reliability of the ReadiBand has also been shown to have high levels of agreement [29].

Athletes were asked to complete a sleep diary every day of the three-week preseason training period. Every morning, athletes were asked to report the previous night’s sleep duration (reported in hours and minutes) and their sleep quality on a scale of 1 to 7 (1: very poor, 7: excellent). These data were collected via the wellness questionnaire described below.

#### 2.3.2. Sleep Questionnaires

The Athlete Sleep Behaviour Questionnaire (ASBQ) and the Pittsburgh Sleep Quality Index (PSQI) were collected to assess various aspects of sleep behaviour. The ASBQ is an 18-item survey that includes specific questions about sleeping habits and behaviours common for elite athletes [30]. Each response for the 18 items is summed to provide a global score with a higher global score indicating poorer sleep behaviour. The PSQI is one of the most commonly used subjective sleep measures [31]. The PSQI is a 19-item questionnaire designed to assess sleep quality and disturbances in clinical and non-clinical populations. The questionnaire produces a global score which can range from 0 to 21, with higher scores indicating poorer overall sleep. Typically, both questionnaires ask athletes about sleep habits over the last month; however, for the purpose of this study, the questionnaires were modified to ask about sleep habits over the past 3 weeks. The ASBQ and PSQI were collected at the beginning of the study (Day 1) and then again at the completion of the three-week preseason training period (Day 21). Both questionnaires were required to be filled out at the same time of day and were administered using Google Forms (Google LLC, Mountain View, CA, USA).

#### 2.3.3. Physical Assessments

##### Maximal Strength Testing

Maximal strength was assessed from one-repetition maximum (1RM) testing of the back squat, bench press and weighted chin-up exercises. Prior to testing, athletes undertook a generalised warm-up that consisted of foam rolling, static and dynamic stretching and basic locomotion. Testing protocols used for bench press and squat exercises have been previously described by Schoenfeld [32]. Each participant was required to perform three submaximal warm-up sets consisting of 2–6 repetitions (reps) with progressively larger loads. Athletes then performed sets of 1–2 reps of increasing weight until a 1RM was attained. Weighted chin-up testing followed protocols previously described by Coyne [33]. Athletes performed five bodyweight chin-ups, followed by a set of three, then two reps with increasing external loads. After warm-up repetitions, athletes performed only single repetitions until a 1RM was attained. Three to five minutes of recovery was allowed between attempts. Each maximal effort set was used to predict each athletes 1RM using the equation 1RM = ([102.78–2.78(R)]/100) [34]. This equation has been shown to have a strong correlation between predicted and actual 1RM for bench press (*r* = 0.993) and back squat (*r* = 0.969) [35] exercises. Testing of all maximal strength exercises occurred at baseline and at week 3 during a single session at each time point.

##### Barbell Bench Press Testing Protocol

Athletes self-selected their hand position and were required to lower the bar to the chest lightly touching before vertically pushing the bar until the arms were fully extended. Athletes could not bounce the bar off their chest, display excessive back arching or receive any help from spotters [32].

##### Back Squat Testing Protocol

Athletes self-selected their hand and foot position and were required to descend until the upper thigh was below parallel with the floor (below 90°). Once the athlete had achieved adequate depth, they were required to ascend to the standing position without any assistance. Depth of the squat was visually assessed by the strength and conditioning staff, and athletes were allowed to wear weightlifting belts, but no other supportive equipment was allowed [32].

##### Weighted Chin up Testing Protocol

Athletes were required to pull vertically until their chin was over the height of the bar and were required to display a controlled return to the starting position. Athletes were not permitted to utilise their legs or any swinging motion, push off the floor to assist or create any elastic energy to utilise. External weight was added by attaching weight plates to the athletes via a weight belt and chain secured around the hips [33]. The external weight added was dependent upon the strength of each individual athlete. The total 1RM weight was established by summing the athlete’s body weight (kg) and weight added.

##### Speed Testing

Speed was measured over both 5 m and 10 m from a standing start position. The athletes were required to perform 2 to 3 trials of maximal effort sprints. Athletes were instructed to stand 50 cm behind the first timing gate prior to starting [36]. Sprint speed was measured using infrared timing lights (Fusion Sport, Brisbane, Australia). The above protocol has been previously established for 10-m trained athletes with intra-trial reliability of r = 0.86 [37]. Speed testing was conducted at baseline and at week 3 within a single session at both time points. All tests were conducted indoors on artificial turf. Prior to testing, participants were able to complete their own individual self-selected warm-up, followed by a standardised warm-up, which consisted of light jogging, short accelerations and dynamic stretching.

##### Aerobic Fitness

Aerobic fitness was assessed at three time points of the study using the Bronco shuttle test. The Bronco test is widely used in rugby environments and consists of running 1200 m in a shuttle (out and back) manner [38]. Each athlete completed a 20-m shuttle, followed by a 40-m shuttle, then a 60-m shuttle. Following the completion of the 60-m shuttle, the participant is considered to have completed one repetition. Participants are required to complete five repetitions as quickly as possible without any rest. The test was performed at baseline, after the three-week preseason training phase, and following the two-week maintenance phase of training. Each test was completed on the same standard-sized rugby union field at every time point (Baseline test = 23 °C, 60% relative humidity (RH), at week 3 = 20 °C, 74% RH, at week 5 = 19 °C, 58% RH). Handheld stopwatches were used to record finishing times for each participant. Prior to testing, participants were able to do their own individual self-selected warm-up, followed by a standardised warm-up which involved run-throughs over 40 m at an increasing tempo (60, 70 and 80% effort), dynamic stretching and change of direction activities at an increasing tempo (60, 70 and 80% effort). Before each test, the participants were reminded of the test protocols and were instructed to give maximal effort throughout the whole test. Aerobic fitness was assessed at baseline, at week 3 and at week 5. The typical error of estimate (TEE) for the Bronco test in elite rugby union athletes is 3.02 s, with a coefficient of variation (CV) of 1.0% (unpublished observations from our laboratory).

##### Body Composition

Bodyweight and skinfold measurements were obtained as measures of body composition for the current study. Body weight was measured to the nearest 0.1 kg using digital scales (Wedderburn, New Zealand). Skinfolds were assessed at eight sites following the protocols of the International Society of the Advancement of Kinanthropometry and summed for the analyses [39]. Bodyweight and skinfolds were obtained at the beginning of the study, following the 3-week preseason training period and after the two-week maintenance training period. All skinfold and body weight measures were assessed by the same accredited professional. Assessment of body composition took place at baseline, at week 3 and at week 5 during a single session. The TEE for the sum of 8 skinfold test in elite rugby union athletes is 4.1 mm, with a coefficient of variation (CV) of 4.7% (unpublished observations from our laboratory).

##### Wellness Assessments

A wellness questionnaire based on the recommendations of Hooper and Mackinnon [40] was completed on the mornings of all training days for the three-week preseason training period. The wellness questionnaire comprised of five questions that included ratings of fatigue, general muscle soreness, general stress, sleep quality and mood. The questionnaire used a 1 to 7 Likert-type scale where 1 represented a low score (e.g., relaxed) and 7 represented a high score (e.g., high stress). A total wellness score was calculated for each athlete ranging from 5 to 35, with lower scores being considered to represent greater wellness.

##### Training Load

Locomotion activity was measured during all on-field team training, units and speed sessions throughout the 3-week preseason phase using an 18 Hz GPS unit (Apex Pro Series Pod, STATSports, Belfast, UK). Each unit was worn on the upper back between the scapulae. To decrease variability, the same GPS unit was used by each participant for all sessions. After the completion of each session, the raw data were downloaded and analysed using the company’s software (Sonra software, STATSports, Belfast, UK).

### 2.4. Statistical Analyses

All descriptive statistics are reported as means ± 95% confidence intervals unless otherwise stated. The data for sleep measures, changes in performance, wellness scores and training load were pooled, and Pearson’s correlations were used to determine if any relationships were present between sleep, changes in performance, markers of subjective wellness and training load. Correlations were interpreted using thresholds of <0.1, *trivial*; 0.1–0.3, *small*; 0.3–0.5, *moderate*; 0.5–0.7, *large*; 0.7–0.9, *very large*; and 0.9–1.0, *almost perfect*. The data were then stratified into two groups based on the midpoint/median for total sleep time (7.5 h). For the purpose of analysis, total sleep time was selected due to being the most accurate and important measure of sleep that was collected via wrist actigraphy [41]. Participants that slept less than 7.5 h on average per night were classed as the low sleep group (LOW, *n* = 15), and participants who slept greater than 7.5 h per night on average were classed as the high sleep group (HIGH, *n* = 14). Statistical analysis was performed using the Statistical Package for Social Sciences (V.26.0, SPSS Inc., Chicago, IL, USA), with statistical significance set at *p* < 0.05 for all analyses. A two-way mixed ANOVA, with 2 (group: HIGH, LOW) × 3 (time: Week 1, Week 3, Week 5) factors was performed for all physical performance measures and sleep questionnaires. Analysis of the studentised residuals was verified visually with histograms and by the Shapiro–Wilk test of normality. The presence of outliers was assessed via inspection of boxplots. Mauchly’s test of sphericity was used to establish equal variance within-subjects for all variables (*p* > 0.05). The main effects were run to identify where statistically significant differences existed. When the main effects were found, post-hoc analysis was performed. Additionally, a Wilcoxon signed-rank test was performed to determine if there were differences in individual scores for sleep questionnaires. Effect-size statistics were calculated using Cohen’s *d* and interpreted using thresholds of 0.2, 0.5 and 0.8 for *small*, *moderate* and *large,* respectively [42]. An effect size of <0.2 was considered *trivial*, and the effect was deemed *unclear* if the 95% confidence interval overlapped the thresholds for both positive and negative effects [43].

## 3. Results

The Pearson’s correlation correlational analyses revealed no significant correlations (*p* ≥ 0.05) between sleep and wellness measures or performance markers, with all r-values in the *trivial*–*moderate* range, as seen in Table 1. As a result, we stratified our data into two groups, (LOW: <7 h 30 min sleep per night, *n* = 15) and HIGH: >7 h 30 min sleep per night, *n* = 14) for all remaining analyses. Differences observed between HIGH and LOW sleep groups for average nightly sleep duration, weekly total sleep duration and overall total sleep duration are shown in Table 2.

Results from the 2-way mixed ANOVA showed that there was a significant group x time interaction for skinfolds, *F*(1,27) = 7.89, *p* < 0.01 and Bronco performance, *F*(1,22) = 7.60, *p* = 0.01, but not for any other performance measure (*p* > 0.05). The HIGH group displayed significantly greater reductions in skinfold measurements at Week 3 (*p* < 0.01, Table 3) and Week 5 (*p* = 0.02, Table 3) compared to the LOW sleep group. The difference in the magnitude of change in the sum of 8 skinfolds between HIGH and LOW sleep groups was 5.4 mm at week 5, which is greater than the TEE (4.1 mm) of the test from our unpublished observations.

Additionally, the HIGH sleep group displayed significantly greater improvements in Bronco performance at Week 3 (*p* = 0.02, Table 3) when compared to the LOW sleep group. The difference in Bronco performance improvements between HIGH and LOW sleep groups was 3.9 s at week 3, which is greater than the TEE (3.02 s) of the test from our unpublished observations.

Sleep questionnaire analysis revealed a significant group x time interaction for ASBQ global scores, *F*(1,27) = 6.39, *p* = 0.01 (Table 4) with the HIGH sleep group showing significant improvement (larger reduction in global score) compared to the LOW sleep group. Additionally, no group x time interactions were found for PSQI global scores between sleep groups (Table 4.). The Wilcoxon signed-rank test displayed significantly different question scores from baseline to week 3 for Q3, (z = −2.06, *p* = 0.03) and Q15 (z = −2.06, *p* = 0.03) of the ASBQ in the HIGH sleep group. No significant group x time interactions were observed between groups for any wellness measures. However, a significant increase (*p* < 0.01) in fatigue was observed from Week 1 to Week 3 across the entire cohort.

## 4. Discussion

The aim of this study was to investigate the relationship between sleep duration and changes in physical performance throughout a 5-week preseason training phase in elite rugby union athletes. Key findings indicate that when the group stratified into two quantile groups, those obtaining greater amounts of sleep (>7 h 30 min) resulted in positive changes in aerobic fitness and body composition compared to those who obtained < 7 h 30 min of sleep per night. Additionally, we observed a decrease in weekly sleep duration and increased fatigue from Week 1 to Week 3 for both groups, suggesting sleep duration is negatively impacted during a preseason phase of training.

While this is the first study to evaluate the link between sleep duration and performance metrics during a preseason phase in elite rugby union athletes, previous research has evaluated the efficiency of sleep extension on athletic performance [24,44,45,46,47]. Prior sleep extension research has shown that an increase in sleep duration can positively impact physical and sport-specific performance. Schwartz and Simon [45] showed that extending sleep by 1 h 43 min for one week significantly improved tennis serving accuracy by 6.1% in collegiate athletes. Additionally, Swinbourne and colleagues [24] reported that increasing sleep duration by an hour per night led to a *small* improvement of 4.3% in reaction time performance in elite rugby union athletes. Lastly, Mah and colleagues [47] showed improvements in sprint time and shooting accuracy in collegiate basketball athletes when sleep was extended from 6 h 36 min during a baseline period to 8 h 30 min over a 5–7 week duration. While the present study did not investigate sleep extension, the results revealed that the HIGH sleep group displayed significant physical performance changes and slept longer than the LOW sleep group. Therefore, it could be suggested that the findings from the current study were similar to those previously reported [45,47].

Although speculative, the positive effects observed in Bronco performance may be related to an increased amount of slow-wave sleep (SWS). Increased sleep duration results in an increased amount of SWS [48]; this is associated with higher levels of growth hormone production, which plays an important role in protein synthesis, muscle growth and repair [19,49]. Higher growth hormone levels have been shown to positively affect aerobic exercise capacity, specifically VO_2_ max and work rate [50,51]. Given that Bronco performance heavily relies on aerobic capacity, the current findings support the beneficial effects of obtaining more sleep on aerobic capacity during preseason periods in elite rugby union athletes.

Further, higher growth hormone levels have been linked to elevated levels of resting metabolic rate (RMR) [52]. RMR is the largest component of energy expenditure and therefore influences body composition [53]. The regulation of ghrelin and leptin have also been linked to sleep duration and are important for body composition. As skinfold measurements are highly influenced by energy intake and RMR, the changes in skinfold measurements observed in the current investigation may suggest that obtaining more sleep can lead to more advantageous outcomes in body composition.

No differences were observed between groups for strength or speed throughout the 3-week training phase. Additionally, results revealed that both groups displayed decreases in strength markers at week 3. The observed lack of change in speed performance is contrary to previous results by Mah and colleagues [47], who demonstrated improvements in sprint performance when sleep duration was extended. Discrepancies in findings between this study and the previous study, as mentioned above may be explained in part by study duration. In the study conducted by Mah, the period between baseline testing and retesting was 7 weeks, whereas the present study was just 3 weeks. In the present study, the 3-week period may not have been an adequate duration to reveal improvements in strength and speed performance measures. Additionally, previous research has highlighted that when concurrent training is utilised, increases in running volume can inhibit strength development and cause decrements in strength and power [54,55]. The interference between running volume and strength is potentially due to high levels of fatigue and muscle soreness caused by high eccentric damage due to running [55]. Interference caused by a weekly increase in running load in the current study may explain why we observed an increase in aerobic fitness and a decrease in strength and speed markers across the 3 week period [56].

The decrease in sleep duration of 1 h 13 min less sleep per night from Week 1 to Week 3 observed for both groups is likely due to increased fatigue. Thornton et al. [9]. previously reported similar decreases in sleep duration compared to the current study. Thornton and colleagues showed an increase in training load and decreased total wellness during a 2-week training camp, which was associated with a decrease in sleep duration of 1 h 39 min per night compared to a pre-camp period. We observed a significant increase in fatigue scores and increased training load from Week 1 to Week 3. Increases in fatigue and training load have been linked to greater sleep disruption leading to lower sleep efficiency and sleep duration [8,57]. Therefore, our findings suggest that the decrease in sleep duration seen across both groups may result from increased training loads resulting in increased fatigue.

The current study highlighted a moderate improvement in the HIGH sleep group for the ASBQ questionnaire global score, which suggests that the HIGH sleep group displayed better sleep behaviours during the 3 weeks than the month leading up to the study. No significant differences were seen for either sleep group for PSQI global scores. It should be noted that the current study’s sleep groups displayed global scores of 5 and above at baseline and after Week 3. A global score of 5 or greater has been suggested to indicate that an individual has moderate sleep difficulties in at least 3 areas or severe difficulties in at least 2 areas [31]. Therefore, our data suggest that elite rugby athletes display poor sleep behaviours [31]. These data highlight the necessity for appropriate sleep education in this population to support physical adaptations during preseason periods.

Given the restraints of conducting research on professional athletes, we acknowledge several limitations in the current study. The study was designed to have minimal impact on training, performance and each athlete’s regular routines; therefore, we could only employ an observational study design, with retrospective analysis. Thus, numerous areas were difficult to control. Firstly, we were unable to control training during the 2-week maintenance period (between weeks 3–5, where athletes were not required to come into the club for training), nor were we able to monitor sleep during this period. Athletes were provided with a training program to complete throughout the 2-weeks; however, we had no control over how strictly the athletes adhered to the program. Secondly, we were unable to control numerous aspects of nutrition. Whilst athletes were provided with the same breakfast and lunch during the preseason phase (Weeks 1–3), we were unable to control caloric intake for all meals. An addition to strengthen the study design would have been to measure all physical performance measures at every time point (Week 1, Week 3 and Week 5), allowing comparisons to be drawn across the 5 weeks for all physical performance measures. Future research should employ an experimental design investigating the differences in physical performance outcomes between a short sleep duration group (e.g., <6 h) and a long sleep duration group (e.g., >8 h) during a period of intensified training. Additionally, researchers should investigate ways of preventing the decrement in sleep duration seen during intensified periods of training (e.g., sleep education, effects of light intensity/device use before sleep and the effects of nutritional interventions to enhance sleep).

## 5. Conclusions

The current investigation is the first to show a relationship between sleep duration and changes in physical performance in an elite rugby union environment during a preseason phase of training. The results suggest that, when grouped for sleep duration, athletes who obtained more sleep displayed greater positive changes in aerobic fitness and body composition than athletes who slept less. Additionally, results showed that sleep duration can be negatively affected during the preseason, which is likely due to an increase in training volume and an increase in fatigue. The current study provides some promising results concerning the effects of sleep duration on physical performance throughout the preseason in elite rugby union athletes and therefore warrants further investigation.

## Figures and Tables

**Table 1:** Pearson’s r-values for correlations between sleep variables, changes in performance markers and wellness measures.

	Body Weight	Skinfolds	1RM Squat	1RM Bench	1RM Chin-Up	Bronco	5 m Speed	10 m Speed	Fatigue	Muscle Soreness	Stress	Sleep Quality (Self-Reported)	Wellness
Total Sleep time	−0.227 ^S^	−0.237 ^S^	0.068	−0.071	0.206 ^S^	−0.097	0.015	0.202 ^S^	0.033	−0.027	0.092	−0.125 ^S^	−0.007
Sleep Efficiency	−0.149 ^S^	−0.156 ^S^	−0.058	0.133 ^S^	0.121 ^S^	0.215 ^S^	0.388 ^M^	0.267 ^S^	−0.305 ^M^	0.302 ^M^	0.136 ^S^	−0.030	0.197 ^S^
Sleep Latency	−0.037	−0.102 ^S^	0.010	−0.053	−0.058	−0.108 ^S^	−0.349 ^M^	−0.393 ^M^	−0.109 ^S^	−0.151 ^S^	−0.199 ^S^	−0.051	−0.149 ^S^
Wake Episodes	0.128 ^S^	0.179 ^S^	0.114 ^S^	−0.007	−0.137 ^S^	−0.165 ^S^	0.049	−0.049	−0.217 ^S^	−0.227 ^S^	0.136 ^S^	−0.068	−0.096
Wake after sleep onset	0.108 ^S^	0.184 ^S^	0.051	−0.024	−0.101 ^S^	−0.201 ^S^	0.170 ^S^	0.071	−0.229 ^S^	−0.239 ^S^	0.125 ^S^	0.038	−0.074

^S^ = small correlation, ^M^ = moderate correlation.

**Table 2:** Differences in sleep duration as assessed via wrist-actigraphy (mean ± SD) for the HIGH and LOW sleep groups across a 3-week data collection period.

	Sleep Duration Nightly (h:min)	Sleep Duration Week 1 (h:min)	Sleep Duration Week 2 (h:min)	Sleep Duration Week 3 (h:min)	Sleep Duration Overall (h:min)
HIGH	7:49 ± 0:15	63:42 ± 4:25	54:06 ± 1:40	54:03 ± 3:28	171:52 ± 6:40
LOW	6:55 ± 0:22	56:34 ± 5:15	48:01 ± 3:33	48:59 ± 2:41	152:36 ± 8:12
HIGH–LOW	0:54 ± 0:06 ^L^	7:08 ± 3:42 ^L^	6:05 ± 2:08 ^L^	6:04 ± 2:23 ^L^	19:16 ± 5:40 ^L^

^L^ = large effect sizes. HIGH: >7.5 h average per night (*n* = 14), LOW: <7.5 h average per night (*n* = 15).

**Table 3:** Data (mean ± 95% confidence limits) for raw change of physical performance measures between Week 1 and Week 3 and raw changes of skinfold, bodyweight and bronco changes between Week 1 and Week 5 for the HIGH and LOW sleep groups including *p*-values and effect size comparisons between groups.

Measure	Condition	Raw Change Week 1 to 3 (Mean ± CI)	*p*-Value	Effect Size (*d*) ± 95% CI	Raw Change Week 1 to 5 (Mean ± CI)	*p*-Value	Effect Size (*d*) ± 95% CI
Skinfold (mm)	HIGH LOW	−8.9 ± 5.9 −6.0 ± 4.0	0.007 *	0.58 ± 0.65 *moderate*	−11.4 ± 8.0 −6.0 ± 3.6	0.020 *	0.92 ± 0.70 *large*
Bodyweight (kg)	HIGH LOW	0.4 ± 1.6 −0.5 ± 1.2	0.091	−0.70 ± 0.72 *moderate*	0.3 ± 1.7 −0.4 ± 1.2	0.158	−0.53 ± 0.74 *unclear*
Bronco (sec)	HIGH LOW	−5.4 ± 3.02 −1.5 ± 3.11	0.022 *	1.25 ± 0.81 *large*	−7.3 ± 5.9 −3.0 ± 9.2	0.277	0.40 ± 0.72 *unclear*
1RM Squat (kg)	HIGH LOW	−5.1 ± 10.0 −5.0 ± 10.0	0.750	−0.15 ± 0.84 *unclear*	-	-	-
1RM Bench Press (kg)	HIGH LOW	−6.1 ± 4.8 −3.3 ± 6.9	0.284	0.48 ± 0.75 *unclear*	-	-	-
1RM Chin-Up (kg)	HIGH LOW	−6.1 ± 6.6 −5.6 ± 7.0	0.853	0.08 ± 0.74 *unclear*	-	-	-
5 m Speed (sec)	HIGH LOW	−0.01 ± 0.03 0.01 ± 0.03	0.799	0.64 ± 0.98 *unclear*	-	-	-
10 m Speed (sec)	HIGH LOW	0.03 ± 0.05 0.02 ± 0.02	0.400	−0.48 ±1.03 *unclear*	-	-	-

* indicates significant difference between groups (*p* < 0.05). HIGH: >7.5 h average per night (*n* = 14), LOW: <7.5 h average per night (*n* = 15).

**Table 4:** Data (mean ± 95% confidence limits) for pre- and post-global scores for ASBQ and PSQI sleep questionnaires between week 1 and week 3 for the HIGH and LOW sleep groups, including *p*-values and effect size comparisons between time points.

Measure	Condition	Pre (Mean ± SD)	Post (Mean ± SD)	*p*-Value	Effect Size (*d*) ± 95% CI
ASBQ	HIGH LOW	44.5 ± 5.7 41.1 ± 4.9	41.0 ± 5.0 41.8 ± 5.1	0.022 * 0.509	−0.63 ±0.76, *moderate* 0.13 ±0.73, *unclear*
PSQI	HIGH LOW	5.7 ± 2.9 5.0 ± 1.8	5.5 ± 2.4 5.5 ± 2.0	0.671 0.135	−0.08 ±0.75, *unclear* 0.26 ±0.80, *unclear*

* indicates significant difference between groups (*p* < 0.05). HIGH: >7.5 h average per night (*n* = 14), LOW: <7.5 h average per night (*n* = 15).
